# Strategies for the implementation of a nasal decolonization intervention to prevent surgical site infections within the Veterans Health Administration

**DOI:** 10.3389/frhs.2022.920830

**Published:** 2022-08-17

**Authors:** Stacey Hockett Sherlock, Cassie Cunningham Goedken, Erin C. Balkenende, Kimberly C. Dukes, Eli N. Perencevich, Heather Schacht Reisinger, Graeme N. Forrest, Christopher D. Pfeiffer, Katelyn A. West, Marin Schweizer

**Affiliations:** ^1^Center for Access and Delivery Research and Evaluation (CADRE), VA Iowa City Health Care System, Iowa City, IA, United States; ^2^Department of Internal Medicine, Carver College of Medicine, The University of Iowa, Iowa City, IA, United States; ^3^Institute for Clinical and Translational Science, The University of Iowa, Iowa City, IA, United States; ^4^Department of Internal Medicine, Rush University Medical Center, Chicago, IL, United States; ^5^Department of Hospital and Specialty Medicine, VA Portland Health Care System, Portland, OR, United States; ^6^Department of Medicine, Oregon Health & Sciences University, Portland, OR, United States; ^7^VA Portland Healthcare System, Portland, OR, United States

**Keywords:** surgical site infection prevention, implementation science, qualitative methods, infection control, nasal decolonization, veterans, ERIC strategies

## Abstract

As part of a multicenter evidence-based intervention for surgical site infection prevention, a qualitative study was conducted with infection control teams and surgical staff members at three Department of Veterans Affairs Healthcare Systems in the USA. This study aimed to identify strategies used by nurses and other facility champions for the implementation of a nasal decolonization intervention. Site visit observations and field notes provided contextual information. Interview data were analyzed with inductive and deductive content analysis. Interview data was mapped to the Expert Recommendations for Implementing Change (ERIC) compilation of implementation strategies. These strategies were then considered in the context of power and relationships as factors that influence implementation. We found that implementation of this evidence-based surgical site infection prevention intervention was successful when nurse champions drove the day-to-day implementation. Nurse champions sustained implementation strategies through all phases of implementation. Findings also suggest that nurse champions leveraged the influence of their role as champion along with their understanding of social networks and relationships to help achieve implementation success. Nurse champions consciously used multiple overlapping and iterative implementation strategies, adapting and tailoring strategies to stakeholders and settings. Commonly used implementation categories included: “train and educate stakeholders,” “use evaluative and iterative strategies,” “adapt and tailor to context,” and “develop stakeholder interrelationships.” Future research should examine the social networks for evidence-based interventions by asking specifically about relationships and power dynamics within healthcare organizations. Implementation of evidence-based interventions should consider if the tasks expected of a nurse champion fit the level of influence or power held by the champion.

**Trial registration:**

ClinicalTrials.gov, identifier: NCT02216227.

## Introduction

Total joint arthroplasty and cardiac surgery are among the most common operations performed by Veterans Affairs (VA) facilities. Healthcare facility data reported to the Center for Disease Control and Prevention's National Healthcare Safety Network shows that *Staphylococcus aureus* bacteria is the most common cause of adult surgical site infections, and specifically the most common pathogen causing orthopedic (38.6%) and cardiac (27%) surgery infections (1). *S. aureus* surgical site infections are associated with devastating clinical outcomes, including recurrent infections, additional surgeries, and death (2). *S. aureus* surgical site infections are usually caused by the patient's endogenous nasal carriage of the bacteria transferring to their own surgical incision site (3–5). Patients who are nasally colonized with the bacteria are more than twice as likely as non-colonized patients to develop *S. aureus* infection (2). The intent of a decolonization protocol is to eliminate or reduce the bacterial load carried by the patient in the nares. Pre-operative decolonization can reduce the incidence of surgical site infections (6, 7). However, nasal decolonization has been inconsistently adopted, and adherence is variable (8–10).

Our VA Health Services Research & Development Service (HSR&D) Collaborative Research to Enhance and Advance Transformation and Excellence (CREATE) program project “Checklist to Prevent Methicillin-resistant *S. aureus* surgical site infections” supported implementing an evidence-based surgical site infection prevention bundle in the orthopedic and cardiac surgical programs of eleven geographically dispersed VA facilities (11, 12). This bundle included testing surgical patient's noses for *S. aureus* colonization, nasal and body decolonization, and antibiotic prophylaxis. Patients were screened for *S. aureus* nasal carriage during a preoperative clinic visit before total joint arthroplasty or cardiac surgery. Patients who were *S. aureus* carriers were prescribed 2% nasal mupirocin ointment to self-apply twice daily for 5 days and chlorhexidine gluconate soap to use daily for the 5 days before surgery. Patients who were not *S. aureus* carriers were prescribed chlorhexidine gluconate soap to use the day before and the morning of surgery (12). *S. aureus* carriers received antibiotic prophylaxis with cefazolin and vancomycin. This bundle is shown to reduce surgical site infections for patients undergoing total joint arthroplasty or cardiac operations (7, 9). However, qualitative evaluation of the VA CREATE project indicated bundle implementation barriers consistent with previous research, including difficulty utilizing the bundle for patients undergoing emergent surgeries (9, 13). We found that patients had difficulty adhering to the intranasal mupirocin ointment protocol (12). Povidone-iodine is an alternative to mupirocin for nasal decolonization (2, 14–16). Research suggests that one-time intranasal povidone-iodine application before surgery may be easier to implement in the clinical setting than mupirocin (15). As well, a survey found that patients preferred povidone-iodine over mupirocin because it had fewer side effects and a more pleasant feeling (8).

This paper presents results from a subset of three VA facilities that independently initiated implementing pre-operative intranasal decolonization with povidone-iodine instead of mupirocin. This alternate evidence-based surgical site infection prevention intervention aimed to reduce challenging patient-burden steps and overcome other barriers with using mupirocin. The purpose of this subset study was to identify barriers, facilitators, and strategies used for successful implementation or uptake of intranasal povidone-iodine decolonization. Specifically, we assessed the role of nurse champions, including their use of relationships and power as facilitators and strategies for implementation of this intervention. We define power as having the authority and ability to influence behavior, for example buy-in from the end users of the intervention. Power also includes authority to make decisions about intervention steps.

Most implementation strategies are complex processes and are socially mediated, involving dynamic interactions among stakeholders (17, 18). Implementation strategies intersect and address contextual determinants, for example, surgical setting or established openness to change at a healthcare facility. Strategies often make use of formal and informal social networks (19). Implementation efforts are facilitated by the involvement of individuals with local influence or power. These individuals may be considered champions, opinion leaders or other change agents (20, 21). Drawing from the field of implementation science, champions are defined as “…individuals who dedicate themselves to supporting, marketing, and driving through an implementation, overcoming indifference or resistance that the intervention may provoke in an organization” [(22), p. 9]. A champion may be formally assigned the role, or individuals may self-identify as champions through their work to support implementation efforts. A champion exhibits strong support for an intervention and drives the implementation of the intervention at their facility. Champions strategically leverage relationships for implementation efforts. They often need to overcome resistance and build support from others (23). Ritchie et al. note that “Having a champion may be necessary (though not sufficient) for successful implementation” [(23), p. 140]. Research demonstrates that nurses are particularly well-positioned to take on the role of champion, and to facilitate the adoption of evidence-based interventions among their peers (24–26).

## Methods

### Setting and sample

Our qualitative study design included semi-structured, open-ended interviews and site visits (27). The three facilities selected for this project were part of the larger eleven-site VA CREATE network. The sub-set of facilities were identified because they had either already switched from using mupirocin to using povidone-iodine (*n* = 1) for nasal decolonization, or key leaders at the facility expressed a goal to switch to using povidone-iodine (*n* = 2). The facilities each independently initiated the intervention locally, not through external facilitation. Two of the three VA facilities (site A and site B) were able to implement the surgical site infection prevention evidence-based intervention and move into the sustainment phase. Sites A and B each had a primary nurse champion responsible for driving through the implementation, and also had secondary nurse champions who helped support implementation activities. The third site (site C) did not have a sustained champion and was unable to move beyond the pre-implementation phase for the povidone iodine intervention.

### Data collection

The principal investigator and the qualitative lead, from fields of epidemiology and anthropology, conducted semi-structured interviews in-person (11 interviews) and by telephone (2 interviews) during 2019–2021. Interviews were conducted with surgery and clinic staff (e.g., nurses, physicians, care managers), infection control staff, and administrative leadership. The qualitative lead and the principal investigator designed the interview guide based on their previous research with the surgical site infection prevention topic and based on previous interview experience with relevant stakeholders. The interview guide focused on learning about the povidone-iodine implementation process, including timeline, decision makers, barriers, facilitators and strategies (see Supplementary material 1 for the interview guide). We utilized a purposive non-probability sample to identify and recruit participants involved with the implementation process or carrying out steps of the intervention (28, 29). The researchers worked collaboratively with each facility to identify interview participants. During interviews, participants were asked to identify additional people who were involved in the implementation or steps of the intervention. When feasible, these additional people were also recruited for interviews. Interviews were audio recorded on encrypted recorders and were transcribed by trained transcribers. The qualitative lead reviewed transcriptions for accuracy against the original recordings.

The principal investigator and the qualitative lead conducted in-person site visits at two of the three VA facilities and telephone interviews with the third VA facility. We collected detailed field notes from observations of infection control practices, surgery-patient flow through the facility, and informal conversations with healthcare workers involved in the implementation (30). At one facility, the principal investigator and qualitative lead attended multiple infection control committee meetings in order to hear leadership discussion around the effort to implement the intervention. At one facility, the qualitative lead attended and took notes during a nursing-focused training to learn how nurses were trained to use the selected povidone-iodine product.

The study was approved by the VA Central Institutional Review Board and Research and Development Committee at the Iowa City VAHCS. Informed consent was reviewed with all interview participants.

### Data analysis

Interview transcripts were imported into MAXQDA, a qualitative data management and analysis software program (31). Data analysis was built on and strengthened by the knowledge gained from qualitative data previously gathered in our larger VA CREATE study. Our coding and analysis team was multidisciplinary (32), from fields of anthropology, epidemiology, infectious diseases, implementation science, and public health. The analysis team included the researchers who conducted the interviews and site visits. Each member of the analysis team had prior experience conducting qualitative research within the topics of infection prevention or implementation science.

Data was analyzed in an iterative process. There were four main activities of data analysis. First analysis activity: We conducted thematic content analysis using a negotiated, or consensus, approach (33–35). Our interdisciplinary team developed a codebook composed of inductive and deductive themes. We refined the existing codebook from the larger VA CREATE study, adding codes around themes of implementation and how change is facilitated. The codebook was organized by higher-order code categories and sub-codes. Codebook definitions were developed for codes which were inductive or more thematic (e.g., timing, compliance) in order to reach a common conceptual framework (36). Definitions were not developed for factual data codes (e.g., staff title, hospital unit). Transcripts were reviewed and coded independently by each member of the coding team. The team held group analysis meetings to review and discuss coding overlap and divergence, in order to apply the codes collectively and systematically to the data using MAXQDA software. Site visit observations and field notes were shared with the team at group meetings, to give further context for some interview responses. Consensus was achieved on all coding.

Second analysis activity: For this paper, interview segments that were coded relating to roles, hospital units, barriers, facilitators, strategies, and recommendations were evaluated for relevancy as implementation factors. The lead qualitative analyst identified and categorized specific implementation strategies used by facilities. Interview segments were mapped retrospectively to the strategies and organizational categories of the Expert Recommendations for Implementing Change (ERIC) compilation (22, 37, 38). The 73 discrete implementation strategies are organized into a taxonomy of 9 categories based on similarity (37). For example, the parent category “Train and Educate Stakeholders” consists of eleven discrete implementation strategies including (1) conduct educational meetings, (2) conduct ongoing training, and (3) create a learning collaborative. Interview analysis showed that implementation strategies were often grouped or overlapped in utilization, and some interview text segments were mapped onto two or more strategies. The ERIC compilation was developed to support systematic identification and reporting of implementation strategies. Documentation of implementation strategies can facilitate dissemination of best practices (17, 39).

Third analysis activity: Following conceptual content analysis (40, 41), the relative dispersion of the interview data mapped to categories and strategies was then ranked by measure of frequency. Frequency indicates how often an implementation category was discussed during interviews. The total number of interview segments mapped to strategies was used for ranked measurement of frequency.

Fourth analysis activity: In keeping with Proctor et al. (42) we identified the primary “actors,” or the stakeholders who drove or enacted strategies and “temporality,” or phase of implementation process when a strategy was used. Implementation process was considered for three major phases, as defined within the VA Quality Enhancement Research Initiative (QUERI) Implementation Roadmap (43): pre-implementation, implementation, and sustainment. Implementation strategies were then considered in the context of power and relationships as factors that influence implementation (19, 42).

## Results

A total of thirteen healthcare workers from the three VA facilities participated in semi-structured interviews. Interview length ranged from 15 to 64 min, with an average (mean) duration of 33 min. Participants included members of infection control teams and surgical nurses. Interview analysis identified multiple themes around implementation process and factors, including barriers, facilitators, strategies, actors, and hospital units. Two main themes emerged from interview thematic content analysis: (1) Implementation of this evidence-based surgical site infection prevention intervention was successful when nurse champions drove the implementation; and (2) Nurse champions leveraged their influence or power along with their understanding of social networks and relationships to help achieve implementation success. Champions used their understanding of decision makers' motivators to facilitate implementation in every phase of the process, by adapting strategies and messages variant on their audience and contextual setting.

### Theme 1: Implementation of this evidence-based surgical site infection prevention intervention was successful when nurse champions drove the implementation

Interview data from all three sites on the topic of how change happens locally, and discussion of the specific details of how the evidence-based intervention came to be implemented, indicated that having a nurse as the champion was a core component (19, 44) to the uptake of the intervention. The ERIC implementation strategy *Identify and Prepare Champions* is one strategy from the larger organizational category “Develop Stakeholder Interrelationships,” and was frequently identified when mapping interview data. Because the champions were also the drivers of most identified implementation strategies, *Identify and Prepare Champions* is the overarching implementation strategy strengthening successful implementation of this evidence-based intervention.

At site A, the nurse champion and their colleagues described the importance of the sustained role and actions of the nurse champion throughout all phases of the implementation process:

“I've been instrumental, and in this evidence-based process, I was involved in an implementation level that was really granular … I did all the research. And then in little bits I did a lot of it [the implementation steps] and then it sort of culminated… I have continued the project and have been tweaking and working on compliance a lot.” -*Primary Nurse Champion, site A*

“I look for [name of nurse champion]'s out there for the interventions and she just did a stunning job. I can't say enough about her project.” -*Infection Prevention nurse, site A*

“Oh yeah, [infection prevention] it's a process, but having someone just, keeping on, [name of nurse champion] she keeps on with it.” -*Infectious Disease physician, site A*

At site B, the nurse champions described adjusting approaches and strategies to drive the process and work through barriers. A primary nurse champion initiated each step of implementing the evidence-based intervention:

*Primary Nurse Champion*: “I'm positive about [the evidence-based intervention]. It's, I mean, it probably took [name of secondary nurse champion] and I–was it a year? Year and a half? Almost in that range to get moving forward, but that's because we had to start from ground zero.”

*Secondary Nurse Champion*: “We didn't have anything to start. I mean, literally ground zero. Nothing, just—”

*Primary Nurse Champion*: “Yeah, and we tried talking to all the outside facilities that had pieces of this or didn't, so we had [facility W]. We had [facility X, Y, Z]. I mean—”

*Secondary Nurse Champion*: “But nobody had the full package. And everybody's VA functions so differently.”

*Interviewer:* “You are making this change happen. Who else would you put in a category like that...?

*Secondary Nurse Champion, site B:* “…I feel like the majority of the work actually has been done by [name of primary nurse champion], and I've just assisted.”

At site C, a member of infection control leadership described achieving some early buy-in during the pre-implementation phase, by utilizing evidence-based data and appealing to a common goal:

“Some of the things that I think were helpful were finding a common goal to organize around and say ‘yes, we all agree, we want lower surgical site infection rates.' This was I think a strong argument [to make] for the surgeons…‘I have data to show you this causes less surgical infections' and that really resonated with them.” *-Hospital Epidemiologist, site C*

However, the infection control member was a local leader facilitating buy-in, and was not the role intended to carry out the implementation steps. At site C when the nurse champion left the facility, the project stalled at pushback during this pre-implementation phase. In the absence of a nurse champion, the early buy-in gained from surgeons was not sufficient support to move past concerns about potentially increasing staff workload:

“…the biggest champion [left the hospital facility]– this was a person who was both in their position and their voice, a strong advocate for it, and that left the opposers…and they said ‘We're not going to implement that. That's not our thing, it's more work and you're not going to give us more staff so we're not going to implement it.”' *-Hospital Epidemiologist, site C*

### Theme 2: Nurse champions leveraged their influence or power and their understanding of social networks and relationships to help achieve implementation success

Another core component to the implementation process was that the nurse champion role needed to have power or authority to make decisions about the intervention details, and power to facilitate necessary approvals from facility leadership or committees. Power may be designated through assignment of status, as in site A where the project champion role was assigned to a motivated nurse:

“… she wanted a meaningful project. And I said well, I think we have one if you really, really REALLY want a meaningful project… I was going to need to mount the energy to do this somehow, so it was very fortuitous that, you know, a fabulous Masters student needed a project. And she had a relationship with all of those people. So it was, you know, I think the stars aligned that somehow it does–, -*Infection Prevention nurse, site A*

Or, power may come from specific role responsibilities within a facility, such as at site B when the primary nurse champion already had an established role tracking infection rate data. The champion was able to leverage the authority of their formal role to enforce the need for a new surgical site infection prevention intervention and achieve early buy-in:

“We had noticed that our infection [rate data] stayed above the national level for the past year and a half, which is considerably high. I reviewed outside facilities within our [region] and we were noted to be the highest. So that really pushed us over the edge of implementing a new protocol.” *-Primary Nurse Champion, site B*

Champions also identify situations where it is strategic to leverage the structural hierarchy of power within a facility, in response to resistance:

“…I just go up the chain…it's very strategic to say, ‘ok, Dr. [Hospital Epidemiologist]or [Infectious Disease physician], you know, they [detractors] need to hear this from you. Not me. I'm just a nurse, you know? So, I'm gonna use your voice and here's what we're gonna accomplish', and so they're incredibly supportive and you know, they're very, very busy so I use that judicially and I think that also helps. ‘Cause when I bring them, we really mean it.” - *Infection Prevention nurse, site A*

### ERIC taxonomy: Implementation strategies and categories utilized by facility stakeholders

In order to document implementation strategies used for this evidence-based intervention, interview data from all stakeholders was mapped to the strategies and categories of the ERIC compilation (see Table 1). Site A and Site B each implemented the intervention. Each site utilized a wide range of implementation strategies, each mapping to 34 strategies. At Site C, the noted strategies were far fewer at 9 strategies. At site C most strategies were linked to the initial discussions at the facility to attempt to gain buy-in for the intervention.

**Table 1:** Interview data mapped to implementation strategies and categories.

	**Implementation strategies**	**Site A**	**Site B**	**Site C**
	**(22)**			
**Categories for** **implementation**	**Strategy**	**Present**	**Present**	**Present**
**Strategies (37)**		**+/–**	**+/–**	**+/–**
Adapt and tailor to context	Promote adaptability	+	+	–
	Tailor strategies	+	+	–
	Use data experts	–	–	–
	Use data warehousing techniques	–	–	–
Change infrastructure	Change accreditation or membership requirements	–	+	–
	Change liability laws	–	–	–
	Change physical structure and equipment	+	+	–
	Change record systems	+	+	–
	Change service sites	–	–	–
	Create or change credentialing and/or licensure standards	–	–	–
	Mandate change	+	+	+
	Start a dissemination organization	–	–	–
Develop stakeholder interrelationships	Build a coalition	+	+	+
	Capture and share local knowledge	+	–	–
	Conduct local consensus discussions	+	+	+
	Develop academic partnerships	+	–	–
	Develop an implementation glossary	–	–	–
	Identify and prepare champions	+	+	+
	Identify early adopters	–	–	–
	Inform local opinion leaders	+	+	+
	Involve executive boards	+	+	–
	Model and simulate change	–	–	–
	Obtain formal commitments	–	–	–
	Organize clinician implementation team meetings	–	+	–
	Promote network weaving	+	–	+
	Recruit, designate, and train for leadership	+	–	–
	Use advisory boards and workgroups	–	–	–
	Use an implementation advisor	–	–	–
	Visit other sites	+	+	–
Engage consumers	Increase demand	–	–	–
	Intervene with patients/consumers to enhance uptake and adherence	–	+	–
	Involve patients/consumers and family members	–	+	–
	Prepare patients/consumers to be active participants	+	+	–
	Use mass media	–	–	–
Provide interactive assistance	Centralize technical assistance	–	–	–
	**Facilitation** ^*^ Nearly all strategies utilized in this table also fall under this Facilitation strategy	**+**	**+**	**+**
	Provide clinical supervision	+	–	–
	Provide local technical assistance	–	–	–
Support clinicians	Create new clinical teams	–	–	–
	Develop resource sharing agreements	–	+	–
	Facilitate relay of clinical data to providers	+	–	–
	Remind clinicians	+	+	–
	Revise professional roles	+	–	–
Train and educate stakeholders	Conduct educational meetings	+	+	–
	Conduct educational outreach visits	–	+	–
	Conduct ongoing training	+	+	–
	Create a learning collaborative	+	–	–
	Develop educational materials	–	+	–
	Distribute educational materials	+	+	–
	Make training dynamic	+	+	–
	Provide ongoing consultation	+	+	–
	Shadow other experts	–	+	–
	Use train-the-trainer strategies	–	–	–
	Work with educational institutions	–	–	–
Use evaluative and iterative strategies	Assess for readiness and identify barriers and facilitators	–	+	–
	Audit and provide feedback	+	+	+
	Conduct cyclical small tests of change	+	–	–
	Conduct local needs assessment	+	+	+
	Develop a formal implementation blueprint	+	+	–
	Develop and implement tools for quality monitoring	–	–	–
	Develop and organize quality monitoring systems	–	–	–
	Obtain and use patients/consumers and family feedback	–	–	–
	Purposely reexamine the implementation	+	–	–
	Stage implementation scale up	+	+	–
Utilize financial strategies	Access New Funding	–	+	–
	Alter incentive/allowance structures	–	–	–
	Alter patient/consumer fees	–	–	–
	Develop disincentives	–	–	–
	Fund and contract for the clinical innovation	–	–	–
	Make billing easier	–	–	–
	Place innovation on fee for service lists/formularies	–	–	–
	Use capitated payments	–	–	–
	Use other payment schemes	+	+	–

The gray values indicate the presence of a strategy at a specific site. The + cells are gray. On the LEFT side of the table, in the original table every other/alternating categories were shaded gray to visually distinguish the separate categories and strategies within.

The relative dispersion of the interview data mapped to the ERIC compilation was ranked by measure of frequency (see Table 2). The top four implementation categories most frequently mapped to interview segments were: (1) Develop stakeholder interrelationships, (2) Use evaluative and iterative strategies, (3) Adapt and tailor to context, (4) Train and educate stakeholders. The first of these, Develop stakeholder interrelationships, comprised 1/3 of the interview segments and the first three of these together comprised 2/3 of the interview segments mapped to implementation strategies/categories. The strategy *Facilitation* from the implementation category Provide interactive assistance was synchronous with nearly all other strategies. We removed *Facilitation* from the measure of frequency table (Table 2) so that it did not overpower the details of the ranking. The frequency rankings identify patterns that offer insight into which categories of implementation were important to the interview participants, when describing the implementation process.

**Table 2:** ERIC taxonomy: ranked frequency of implementation strategies and categories utilized by facility stakeholders.

**ERIC implementation strategy organizational category**	**Frequency ranking (i.e., *how often***
	**were strategies within category discussed)**
^*^Develop stakeholder interrelationships	1st
^*^Use evaluative and iterative strategies	2nd
^*^Adapt and tailor to context	3rd
Train and educate stakeholders	4th
Change infrastructure	5th
Engage consumers	6th (tie)
Utilize financial strategies	6th (tie)
Support clinicians	8th
Provide interactive assistance ^**^ranking based only on presence of the *Clinical Supervision*	9th
strategy; *Facilitation* strategy was synchronous for nearly all documented strategies	
^*^ 2/3 of the quote segments were mapped to the top three ranked categories for frequency	

### Develop stakeholder interrelationships: Category and discrete strategies

The ERIC organizational category “Develop stakeholder interrelationships” comprised 1/3 of the mapped strategy interview segments, indicating it was of particular salience to the implementation and setting of this intervention. A theme of the interviews was the importance of building initial and ongoing buy-in and motivation from stakeholders to facilitate the intervention. Champions at Site A and B described efforts to develop and sustain these relationships with stakeholders that would need to carry out steps of the intervention:

“When you're looking at process improvement, you know, your process is only as good as the people you work with.” *-Primary Nurse Champion, site A*

One of the implementation strategies within this category is *Build a Coalition*, defined as “Recruit and cultivate relationships with partners in the implementation effort” (22), and was utilized by nurse champions at sites A and B during the pre-implementation phase, and into the implementation phase.

One nurse champion described how they intentionally prepare different “elevator pitches” for different stakeholders. This effort serves to create a coalition of staff from different settings and roles, all with buy-in for the evidence-based intervention:

“When I talked to people about change…I always wanna know, where's the motivation? How do I sell this to you? …I always like to really drill down to *why* and I will walk in and I have an elevator speech for every *why*…you want to light that fire in people. And you want them to feel that urgency to participate.”*-Primary Nurse Champion, site A*

Early involvement during pre-implementation allowed clinic staff, who are the end users of the intervention, to become invested in creating an intervention appropriate for the setting and patients. This involvement let staff know their feedback was valued. Involvement of clinic staff during pre-implementation also facilitated future sustainment of the intervention, as the staff who work directly with the patients can offer expertise that can facilitate patient adherence. Further, building a coalition facilitated buy-in through letting clinic staff know they were not expected to make the intervention work on their own.

“And helping with those [instruction handout] tools for the patient and for the nurse– [the primary nurse champion] made sure she invited everybody she could to those meetings to help edit them and to get as many eyes on them as possible... I think they [staff] appreciated they could put in some input. But didn't have to *do* the project, which is understandable.” -*Secondary Nurse Champion, site B*

### Other ERIC strategies and influence of relationships

Additional categories and strategies utilized for this intervention intersect with building relationships and understanding of social networks. The category “Use evaluative and iterative strategies” was ranked 2^nd^ in use for measures of both depth and breadth. From this category, the implementation strategy *Audit and Provide Feedback* was a core component during each implementation phase at both site A and B. At site A, the Nurse Champion evaluated ways to share audit data with the clinic staff regarding if the intervention was being used correctly, and determined that performance feedback would be best received when coming from a peer in the operating room (OR) setting:

“…what has been really helpful is that...the OR nurse does this audit and then she goes to her peers and she reminds them [to do the intervention]. ‘Cause, you know, I'm the…PACU [post-anesthesia care unit] nurse, I'm the CNL [clinical nurse leader], I'm someone who keeps telling them they did something wrong, but I don't really have a relationship with them as much…when they need education, I come, and I give the education. And my way may not be the OR way. I've never been an OR nurse. So, you know, they kinda… speak their own language, they have their own culture…” *-Primary Nurse Champion, site A*

At sites A and B, the nurse champions utilized the implementation strategy *Stage Implementation Scale Up*, also from the “Use Evaluative and Iterative Strategies” category. At site B, the champions purposely phased the povidone-iodine bundle implementation by launching with surgery services that they knew would be open to change rather than starting with the services where the surgical site infection reduction had greatest immediate need:

“And one of the reasons we picked these two services is just because they don't mind change. They were agreeable to it. So rather than jumping it right to general surgery… it wasn't that we had most infections in these populations at all. We just picked them because they were the easiest to work with at the time.” -*Secondary Nurse Champion, site B*

“Ortho did it ‘cause we were hoping they could get away from having to use mupirocin,” -*OR Nurse Manager, site B*

At both sites A and B, after rollout with targeted services, an understanding of comfort level and feedback from clinic staff allowed for quick scale up to additional surgery services:

“And we quickly moved it to all of the surgeries because it simplified the intervention within that pre-op clinic.”*-Infection Prevention nurse, site A*

“I think it was good doing it with one or two services just because then you work through the issues and the questions people had…I felt like they [clinic staff] kinda– got their feet wet. They felt confident so when they were rolling it out needing to do it at a faster pace or because the volume was higher, they were more comfortable with it.” *-OR Nurse Manager, site B*

### Implementation strategies: Actors and phases

**Figure 1:**
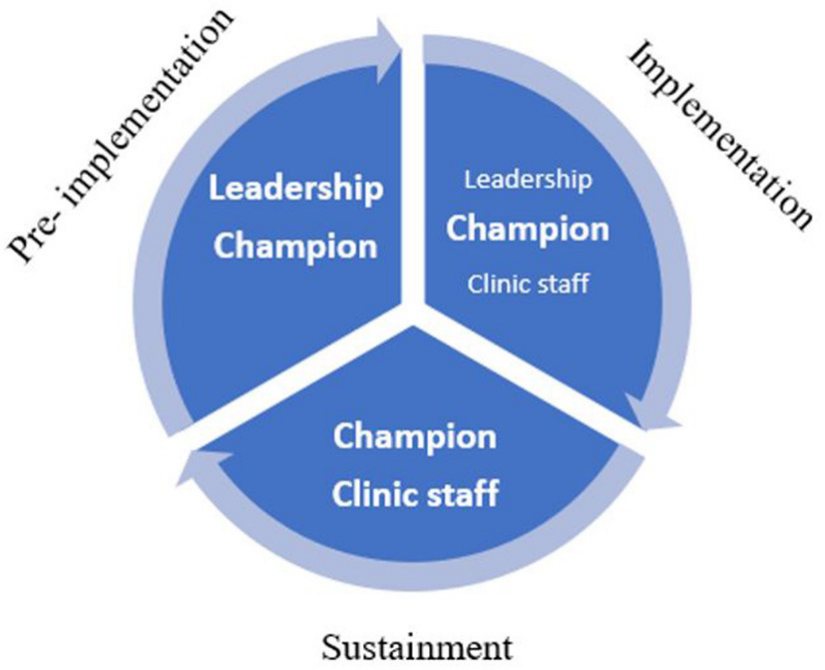
Actors/drivers of implementation strategies and phases of implementation.

Implementation strategies were driven by different actors or stakeholders during different phases of the implementation cycle (see Figure 1).

Nurse champions were actors or drivers of implementation strategies across all three phases, and in collaboration with leadership and clinic staff. Many of the strategies driven by nurse champions took place during the implementation phase. Facility leadership primarily used implementation strategies during pre-implementation. Clinic staff tended to be involved minimally during pre-implementation and were more involved with utilizing implementation strategies in the late-implementation and sustainment phases.

Implementation strategies used by leadership largely focused on achieving buy-in for the intervention, and centered on utilizing the ERIC strategies *Inform Local Opinion Leaders* and *Involve Executive Boards*, both of which fall into the parent category “Develop Stakeholder Interrelationships.” Stakeholders from each of the three sites discussed the importance of gaining buy-in from facility opinion leaders who hold influence and power, which can facilitate moving forward in the face of pushback:

Interviewer: “Who are the key decision makers for saying we're…gonna make this change?” Respondent: “Probably [Infection Prevention nurse], [Infectious Disease physician], myself, Chief of Surgery, the pre-surgical clinic, the nurse manager…everybody plays probably some semi-major role. We had a couple of services within surgery that gave us some pushback, said ‘I don't know if we need to do this'…but I think the Chief of Surgery was fairly strongly behind it, so it doesn't make sense not to standardize this. And so, I think that he was helpful in getting it done for sure.” *-Hospital Epidemiologist, site A*

Several interviews highlighted the observation that just one or two influential people can derail or halt implementation, described by leadership at site A as being “bogged down by…organizational constipators”:

“Change can be so hard. I have been a part of several changes here and it's amazing how one or two people can really be that barrier.” -*Nurse care coordinator, site A*

At site B, the implementation process timeline was influenced by pushback from the OR nurse manager, who was protective about the workload and workflow for the staff they manage:

“I'll be very frank. I think when we first introduced this it was like, ‘oh God just here's another thing I've gotta do'… I know I kind of pushed back ‘cause I just—‘don't they know it's just another thing we gotta do?' It's busy work.” *-OR Nurse Manager, site B*

After the intervention was well-established with fidelity, the same nurse manager shared they had changed their mind about the burden of the evidence-based intervention and felt acceptance for using it within their unit:

“…I was probably one that was worried that it was gonna add a lot of time to preparation. And I'm delightfully surprised that it did not. They [nurses] found ways to work it into their normal assessment, pre-op process, that it hasn't added a lot of time. Much less than what I thought it might be. I tend to be kinda protective and try to think about- value their [nurses] time just like everybody else's– but I think overall it's gone really well.” *-OR Nurse Manager, site B*

Once the evidence-based intervention had buy-in from opinion leaders and approval from relevant executive boards or committees, the active use of implementation strategies by those in leadership tended to be reduced but continued at a lower level of engagement into the implementation phase.

Nurse champions maintained steady involvement in all stages of implementation, including utilizing strategies to continue to achieve buy-in and motivation as the intervention moved through the implementation and sustainment phases:

“I think, you know, process improvement, which was what this really is, my part of it, it is a marathon. It is not a sprint. And some days it feels like you're running as fast as you can, but you're still in the marathon and you have continually–, you know when people, like, attack you and say verbally, ‘I don't know why we are doing this. This is stupid,'…so what really got people onboard is bringing back the data and results frequently around every three months for about the first year.” -*Primary Nurse Champion, site A*

At both sites A and B, the nurse champions along with some members of leadership iteratively educated staff at every position level affected by the intervention to achieve and sustain buy-in. During interviews, clinic staff shared that it was influential when they understood how the intervention helped patients. At each site, surgeons and infection prevention staff were shown infection rate data and research that demonstrated intervention effectiveness, in order to facilitate buy-in. While this data was also shared with the nurses who directly use the intervention with patients, one nurse champion described leveraging their comprehension that nurses may be motivated through education and awareness of the impact of surgical site infections on an individual patient level:

“… if you can do a vignette of a patient with a joint infection and their lifestyle, it's really–, that's really impactful.” -*Primary Nurse Champion, site A*

Nurse champions at both sites A and B initiated strategies to formalize and standardize the evidence-based intervention as expected practice, as the intervention moved into the sustainment phase of implementation. At site A, after a one-year pilot of using povidone-iodine, the nurse champion wrote a Standard Operating Procedure (SOP) that formally allowed the povidone-iodine to be a supply and not require a prescription. This SOP “…was signed off by a lot of entities…it's signed off by the chief of surgery, anesthesiology, ID. You know, it's quite a long list.” (*Infection Preventionist nurse, site A)*. Allowing the povidone-iodine to be available without a prescription overcame major barriers that occurred for the mupirocin decolonization product, which had delays and communication issues with the prescription ordering process. At site B, the nurse champions wrote a decolonization bundle policy and a yearly competency intended for inpatient nurses. The formalized materials were planned to simplify and streamline the decolonization process for patients coming to surgery from an inpatient unit, and allow patients to receive the evidence-based intervention bundle while in the inpatient unit. Developing these materials also illustrates the iterative nature of implementation, moving the process back into pre-implementation phase to plan for the expansion of the evidence-based intervention into a different setting within the facility.

Clinic staff were drivers of refining and adapting steps for working with patients and for minimizing their increased workload, as the project moved from implementation into sustainment. Many of these implementation strategies map to the ERIC category “Engage Consumers” and centered around patient education to enhance adherence, and engaging the patient to apply the decolonization products:

“We say [to the patient], ‘Can you do this?' Because it takes a burden off the nurse, ‘cause [the nurse] can do other tasks while [patients] are completing that. And then we go in and help them finish. If they have somebody that's in with them, a spouse, we can let them do it also…But we always make sure and complete the remainder of the process if they can't accomplish what they need…Some people think [the povidone iodine] is unpleasant. They don't want anything stuck up their nose…If we explain it to ‘em, they're ok with that little bit of discomfort to keep the infection at bay.” -*OR Nurse Manager, site B*

### Implementation strategy: Mandate change

At site C where the evidence-based intervention lost the nurse champion, an infection control leader stated that a mandated change or top-down approach initiated by a high level of hierarchical power may be needed for implementation at their facility:

“Another thing of course that's very motivating is a ‘Have To'. So if the oversight committee…or the VA itself were to say ‘you must do this, this is the required perioperative best practice implemented now', then I don't think there would have been any issue, they would have just done it.” -*Hospital Epidemiologist, site C*

While a mandate may kick-off initial steps to implement an intervention, one nurse champion pointed out that mandates are void of context for the nuanced details and decisions that go into implementation:

“The problem with doctors deciding that…it's a good idea for a nurse to do something, is you have to understand the granular level, right? And you have to understand how work flows, and you have to figure out how this one puzzle piece fits into other people's workflow ‘cause if you just come and, say,”…the change needs to happen, you figure it out”– You have to have someone who's gonna say, “All right, we're gonna figure this out together. We're gonna support you,” and that sort of thing and I think that that goes a really long way.” *-Primary Nurse Champion, Site A*

### Implementation strategy: Assess for readiness and identify barriers and facilitators

The implementation strategy *Assess for Readiness and Identify Barriers and Facilitators* is defined as “Assess various aspects of an organization to determine its degree of readiness to implement, barriers that may impede implementation, and strengths that can be used in the implementation effort” (22). Organizational culture related to openness for innovations can be an aspect or contextual factor that impedes or strengthens readiness for change.

Interview respondents identified their local facility culture or openness to change as a key factor for the readiness to embrace the implementation process. At site A, several stakeholders talked about openness to change as a historical facilitator:

“[site name], you know, historically– I mean, you probably hear this from others, is that we've embraced…change and prided ourselves as a facility that we're kind of on the, you know, good side of new innovations and stuff. And so, if you kind of come into a place that's in an environment like that, then that's easier to get things done obviously, and the staff is more used to changes and initiatives and improvement projects and all that stuff.” *-Hospital Epidemiologist, site A*

This facility-wide embracement of change is in contrast to site B. Nurse champions felt that historically, local efforts to implement change are siloed or given to one or a few people to implement, without the feeling of facility-wide openness to change:

“I don't really feel like there's been a[n] overwhelming certain person that has helped with this at all…And that's what our culture is though. And I don't know if other VAs are like this though. I feel like that's just the way things are here. You know, if infection prevention does it, well then THEY just do it. Or it's very siloed and not a lot of people jumping on board. We can't get a lot of people back onto the wagon, you know. It's kind of a lonely wagon.” – *Secondary Nurse Champion, site B*

## Discussion

In this study, we evaluated the real-world implementation of decolonization interventions at three VA facilities. We did not impose, develop, or facilitate use of implementation strategies; rather, we conducted qualitative research during phases of implementation in order to identify and document the implementation strategies used by stakeholders. Identifying the implementation strategies utilized to improve the uptake of evidence-based interventions is critical. Documenting implementation strategies that were successful at one site may facilitate implementation in similar settings. Yet, there may not be a clear replicable roadmap that every site can use without adaptation for local contextual factors.

One strategy, *Identify and Prepare Champions*, strengthened implementation success. However, it was not its impact as a single strategy but how champions were able to choose, test, and merge various implementation strategies to improve uptake of povidone-iodine. In this implementation effort, the champions also acted as facilitators. In addition, our qualitative analysis points toward the importance of how champions *leveraged their influence or power and their understanding of social networks and relationships* as a component of champions' roles and responsibilities. Power by role or by assignment facilitated the role of the champion as a driver of implementation strategies. Results of our research supports the finding by Ploeg et al. (25) that the role of nurse as champion facilitates uptake of evidence-based practices in health care organizations. Depending on setting and contextual factors, there is support for the role of a nurse champion in implementation of an evidence-based intervention for surgical site infection prevention. We found that nurse champions delivered multiple implementation strategies across each implementation phase, and had particular social knowledge of how to achieve buy-in by understanding the motivational factors that influence decision makers and end users. Many actions and steps within the hospital setting are very siloed, and it takes a person with understanding of how patients flow through the facility, how information flows, and the nuances of influence to put all the pieces together. Champions strategically leveraged the structural hierarchy of power within their facility by utilizing local leaders to publicly support the intervention and facilitate buy-in. Understanding of social networks is important: who works with who, who listens to who, what is the hierarchy of power. Ploeg et al. suggest social network mapping could be used to examine the strength of links and ties between facility champions, peers of champions, and leadership or those with authority positions (25).

At Site B, the primary nurse champion was motivated to initiate the intervention when local infection rate data remained high. The champion sought out comparators for infection rate data, which also acted as a source of their commitment to drive the implementation process. Sites A and B each implemented the intervention and moved into sustainment, where site C was not able to implement. Considering the implementation strategies mapped to the ERIC taxonomy, sites A and B each utilized 4 times the number of implementation strategies as site C. This reflects the wide range and number of strategies that may be needed to move an intervention into sustainment. As well, a large number of strategies at sites A and B were driven by the local nurse champion. Site C lost their local champion and in the absence of a champion did not move forward with implementation. Future research may be needed to determine key attributes or preconditions (19) to implementing this intervention in surgery settings.

Regarding the ERIC categories, our findings by measures of both the presence/utilization of the implementation strategies within the categories (Table 1) and the ranked frequency count of interview segments (Table 2) largely align with the top “importance” and “feasibility” mean ratings by category, as conducted by a panel of implementation science and clinical experts (37). It is imprecise to compare the ranking data directly because different approaches were used; Waltz et al. (37) prospectively asked experts for ratings using the list of strategies, and our data was applied retrospectively through interpretation of qualitative interview data. However, this does indicate the potential to identify ERIC strategies to plan implementation approaches, and iteratively track the real-life influence and utility of strategies in order to better understand implementation facilitators. When considered in conjunction with the interview thematic analysis, the table of strategies and the frequency rankings help illuminate the real-world implementation process for this evidence-based intervention.

White et al. (45) conducted a systematic review to identify strategies used in implementation of the World Health Organization Surgical Safety Checklist (SSC). While the systematic review focused on studies from low and middle income countries, the surgical setting, overarching goal related to patient safety, and use of a checklist of steps are similar to the evidence-based intervention of our study. The authors found the five most commonly used categories from the ERIC compilation of strategies were “train and educate stakeholders,” “adapt and tailor to context,” “provide interactive assistance,” “develop stakeholder relationships,” and “support clinicians.” Our study indicated common use of three of these: “train and educate stakeholders,” “adapt and tailor to context,” and “develop stakeholder relationships.” Our study found some use of “support clinicians” and of “provide interactive assistance.” Our study additionally indicated common use of the category “use evaluative and iterative strategies” (see Table 1).

In reflection on the implementation strategies in practice, we note that *Facilitation* was not a distinct stand-alone strategy but by definition as a support process, was combined with other implementation strategies. Perry et al. (46) observe that facilitation is a very broad concept, and suggest an expansion of the definition for the ERIC strategy *Facilitation* to better align with how implementation happens in practice. The current definition is: “A process of interactive problem solving and support that occurs in a context of a recognized need for improvement and a supportive interpersonal relationship” [(22), p. 9]. The proposed broadened definition is: “A multi-faceted interactive process of problem solving, enabling and supporting individuals, groups and organizations in their efforts to adopt and incorporate innovations into routine practices that occurs in a context of a recognized need for improvement and a supportive interpersonal relationship” [(46), p. 5]. We support this proposed definition based on our finding that the nurse champions also acted as facilitators to drive most identified implementation strategies, and that the strategy *Facilitation* was synchronous with nearly all utilized implementation strategies.

The VA continues to support and grow the use of implementation science approaches to prevent healthcare associated infections including surgical site infections (23, 47). Research priorities include identification of barriers, facilitators, and strategies for the implementation of healthcare associated infection prevention practices. The ERIC compilation of strategies is useful because mapping to a taxonomy of named, specified implementation strategies helps improve reporting of effective multilevel implementation processes. Identifying the strategies used for successful implementation is useful for future intervention attempts, as it facilitates replication of the intervention. We recommend targeted, real-time documentation of implementation strategies by those doing the implementing. The Cognitive Walkthrough for Implementation Strategies (CWIS) is a method designed for evaluating complex, socially mediated implementation strategies in healthcare (17). While understanding the process does not set a single roadmap to implementation success, the insight could inform design of future evidence-based intervention implementation and facilitate future replication. It would be valuable to know more about site knowledge of or familiarity with strategies, and how this experience influenced implementation.

## Limitations

Implementation is complex, and context and factors cannot be fully explained in this paper. This paper presents a focus on the relationship and power factors as they interrelate with implementation strategies, actors, and phase of the implementation cycle. Implementation is a dynamic process. The data presented in this paper are a reflective snapshot of what was considered important for implementation on an evidence-based intervention intended to prevent surgical site infections in VA settings. We may have missed identifying implementation strategies that were flexibly applied or briefly applied as barriers arose, or strategies that were applied but less effective. We documented the implementation strategies that stakeholders felt were more important or effective, and this information was triangulated through site visits, observations, and interviews with available stakeholders. Some healthcare staff involved in implementation were unavailable for an interview, so we may not have captured information from all roles involved in the intervention. Participants represent three facilities and this small sample size is a limitation, as it may not account for factors that influence implementation in other settings. We sought out all key stakeholders for interviews, including individuals who were not supportive of the intervention, however this attempt to talk with non-supportive individuals was more purposive at facilities where we conducted site visits because we could more easily access staff in-person than we could reach by telephone.

## Conclusion

Implementation is complex and dynamic, and qualitative data collection and analysis offers insight into the ongoing process through pre-implementation, implementation, and sustainment phases. For this evidence-based intervention, the nurse champions' designated power to make decisions and ownership of the project facilitated strategic approaches to achieving implementation success. Nurse champions drove the day-to-day implementation of this evidence-based intervention to reduce surgical site infections. Nurse champions sustained implementation strategies through all phases of implementation. As well, nurse champions consciously used multiple overlapping and iterative implementation strategies, adapting and tailoring strategies to stakeholders and settings. Findings suggest that nurse champions leverage the influence of their role as champion along with their understanding of social networks and their understanding of motivations based on relationship building to help achieve implementation success. They were able to leverage their relationships with different stakeholders and understanding of the working healthcare system to select strategies and facilitate the intervention. Future research should examine social networks and local social power and hierarchies in planning and carrying out evidence-based interventions by asking specifically about relationships and power dynamics within healthcare organizations.

## Data availability statement

The datasets generated and/or analyzed during the current study are not publicly available due to participant privacy but are available from the corresponding author on reasonable request.

## Ethics statement

The studies involving human participants were reviewed and approved by Human subject review and approvals were sought from the VA Central Institutional Review Board and Research and Development Committee at the Iowa City VAHCS. Written informed consent for participation was not required for this study in accordance with the national legislation and the institutional requirements.

## Author contributions

EP, MS, HR, GF, and CP: study conception and design. SH, MS, and KW: data collection. SH, MS, EB, KD, and CG: analysis and interpretation of results. SH: draft manuscript preparation. All authors reviewed the results and approved the final version of the manuscript.

## Funding

This work was supported financially by the VA-CDC Practice-Based Research Network, which was funded collaboratively by the VA Health Services Research & Development Service (HSR&D) service, the Centers for Disease Control & Prevention (CDC), and the Collaborative Research to Enhance and Advance Transformation and Excellence (CREATE) program (CRE 12-291 to primary investigator EP) from the VA HSR&D.

## Conflict of interest

Author MS reports grant and donated product from PDI Healthcare and 3M for a different study. He reports honoraria for creating educational module on surgical site infections and honoraria for presenting on surgical site infections. The remaining authors declare that the research was conducted in the absence of any commercial or financial relationships that could be construed as a potential conflict of interest.

## Publisher's note

All claims expressed in this article are solely those of the authors and do not necessarily represent those of their affiliated organizations, or those of the publisher, the editors and the reviewers. Any product that may be evaluated in this article, or claim that may be made by its manufacturer, is not guaranteed or endorsed by the publisher.

## Author disclaimer

The views presented in this manuscript are those of the authors and do not necessarily reflect the position or policy of the Department of Veterans Affairs or the United States Government.
