# Supplementary material for: Strategies for the implementation of a nasal decolonization intervention to prevent surgical site infections within the Veterans Health Administration
Source: Front Health Serv. 2022 Aug 17;2:920830. doi: 10.3389/frhs.2022.920830 (PMC10012655; doi:10.3389/frhs.2022.920830)
Supplement: Supplementary file 1 [file Data_Sheet_1.docx]

**SSI 2.0 Interview Guide**

**1. Process Evaluation**

Here is the flow chart of the SSI prevention bundle. Some of the hospitals modified steps to best fit their specific facility. I’d like to walk through the steps and find out what your facility is doing at each step, as well as the barriers or facilitators for each step.

<image of process flow chart>

**At each step ask**:
-What are/were the problems with this step (barriers)?
-What makes/made this step easier (facilitators)?

*[Interviewer Instructions: ask prompts for the steps where the respondent has direct knowledge of the process]*

Pre-op/Surgery eligibility

How many visits?

When take place? How many days or weeks ahead of surgery do visit/s take place?

At what point/who decides surgery is needed?

Screening

When is the patient screened for MRSA?

How is it determined which patients to screen?

-Which types of surgery are screening patients?

-What about emergent surgeries?

-Known MRSA-positive patients are typically flagged in CPRS. Are known positive patients also screened, or automatically treated as positive?

Who puts in the order for the screening?

Who swabs the patient?

How does the swab get to the lab?

What process does lab use?

How long does it take to get the results?

Who receives results from lab?

How are results relayed back from lab?

Decolonization medications

Are the clinics currently using mupirocin or povidone iodine or both?

Who puts in orders (if needed) for CHG? Mupirocin? Povidone iodine?

Is there a separate order for CHG for patients who are negative? (are negative patients still getting their needed CHG per protocol/ night ahead and day of surgery)

Is there a universal order for CHG for surgery patients (all patients getting same amount of CHG)? Which surgeries have universal orders?

For patient education:

1) When are patients educated about the decolonization protocol? (e.g. before results known? only if screen +?),

2) Who educates patients about the medicines?

3) How are they educated? (Paper sheet, in-person, phone call)

When does patient get medicines? And how do they receive them?

Who pays for the Povidone iodine? Patient billed separately for medication? Rolled into surgery cost?

What CHG product is used (wipes, soap, sponges)?

What povidone iodine product is used?

Day of surgery

When does patient come in for surgery?

What is your impression of patient compliance with using the mupirocin?

What is your impression of patient compliance with using the CHG?

Who administers the povidone iodine (Patient self-administer, day of surgery nurse)

Best practice for povidone iodine is to use just prior to surgery, 30 seconds in each nostril, x2. What are your thoughts on implementing this?

When is povidone iodine administered/ timing?

How many times is PI administered before surgery? Any administered after surgery?

Where/who are best people to administer to patient?

Are patients using single-use products? What happens to remaining product?

How is use of povidone iodine logged? Where is it logged?

Who asks the compliance questions? Are they being asked of patient directly or inferred?

What is day of surgery process for cleaning skin?

Which perioperative antibiotics are being used? Universal or based on MRSA screening?

When are orders put in for the perioperative antibiotics?

Who puts in the order for the perioperative antibiotics? Is it a weight-based order?

How is perioperative antibiotic tracked? (examples include: scan barcodes, record in CPRS, record in paper logbook)

How many surgeries a month?

What do you do when a patient arrives and they need a shower? Is there a place for the patient to bathe/shower?

**2. Other Implementation Questions**

We talked about each step and some of the barriers you’ve addressed and changes you’ve made in the process to make this happen. Have there been any other changes to the process that we haven’t covered? Are there changes that we are missing?

Is the process is different from what you thought it would be when planned?

Is there a champion or point person for this bundle?

This bundle crosses over several units. Where do you see steps that a patient could fall through the cracks? What do you recommended to address that?

Are the staff resources adequate to implement this bundle?

Have you had any changes in staff?

Have you had to stop conducting surgeries for any period of time during this intervention?

Have any other SSI prevention interventions been implemented?

Who is tracking infection rates? Which staff receive the data on infection rates?

**Ask everyone:**

How does change happen at your VA facility?

What recommendations do you have for other VAs implementing this bundle?

If this bundle became a national mandate, what would need to happen to make it work? What would make this easier for you?

How can we involve transferring hospitals in decolonization, if patients are evaluated there pre-surgery and then come to main VA for surgery? Can originating VAs screen for MRSA? E.g. at one facility the cardiac patients are screened.
